# Individuals with Psychopathic Traits and Poor Attitudes towards Animals Can Recognise Infant Features But Give Them Reduced Attentional Priority

**DOI:** 10.3390/ani10040721

**Published:** 2020-04-21

**Authors:** Grace A. Carroll, Leah R. Cohen, Aideen McParland, Sam Jack, V. Tamara Montrose

**Affiliations:** 1 Animal Behaviour Centre, School of Psychology, Queen’s University Belfast, David Keir Building, 18-30 Malone Road, Belfast BT95BN, UK; cohen.l@husky.neu.edu (L.R.C.); a.mcparland@qub.ac.uk (A.M.); sjack02@qub.ac.uk (S.J.); 2 Independent Researcher, Manchester M139PL, UK; v.tamara.montrose@gmail.com

**Keywords:** infant features, psychopathy, animal attitudes, eye-tracking, gaze data, cute

## Abstract

**Simple Summary:**

Certain facial characteristics in companion animals are perceived by humans as being ‘cute’. This includes having large eyes, a round head and a small nose and mouth. These characteristics, which are shared with human infants, trigger care-giving responses in humans. Despite this, however, companion animal abuse occurs. The aim of this research was to better understand cognitive processes of people with pro-social personality traits and positive attitudes towards animals compared to those with anti-social personality traits and negative attitudes towards animals. This was done by assessing participants’ ability to detect cues of cuteness in animal and human infant faces (study 1) and by assessing attention to cuteness cues via an eye-tracking task (study 2). Findings indicate that the ability to detect cuteness cues is widespread, regardless of personality or attitudes. However, individuals with anti-social personality traits and negative attitudes towards animals chose to pay less attention to ‘cute’ stimuli in the eye-tracking task. This proof of concept study is an initial step in determining how individuals ‘at risk’ of committing animal abuse process information on infant features in animals.

**Abstract:**

Infant features are physical traits that are characteristic of human infants and include facial features such as large and low-lying eyes, and a small nose and mouth. Animals possessing high levels of infant features elicit care-giving responses in humans. Despite this, animal cruelty is a common occurrence. The aim of this research was to determine whether the ability to recognise and/or attend to infant features is linked to subclinical psychopathic traits and attitudes towards animals. Using a community sample, participants (n = 387) completed a cuteness forced-choice task. Self-reported psychopathy and attitude towards animals were not related to the participants’ ability to detect cues of cuteness in human infants and animals. In a second study, participants (n = 142) were screened for low versus high primary psychopathy and low versus high animal attitude scores. A Psychopathy-Attitude Composite score was created and a subset of participants (n = 50) from the upper and lower quartiles completed a free-viewing eye-tracking task where ‘Cute’, ‘Neutral, ‘Monetary’ and ‘Control’ images were presented in pairs. Higher levels of psychopathic traits and an anti-animal welfare attitude were associated with decreased attention to ‘Cute’ images in terms of decreased dwell time, mean fixation duration and mean fixation count, measures of voluntary attention. There were a number of interactions between Psychopathy-Attitude Composite classification and attention to each image category in terms of dwell time, first fixation duration, mean fixation duration and fixation count. These findings support the theory that individuals with psychopathic traits recognise facial cues of vulnerability but choose to give them reduced attentional priority. This may have implications for animal welfare.

## 1. Introduction

Infant features are physical traits that are characteristic of human infants and include facial features such as a large forehead, large and low-lying eyes, and a small nose and mouth [1,2]. Animals possessing high levels of infant features are perceived as being ‘cute’ and elicit care-giving responses in humans [3]. It is thought that these responses are generalised ‘empathetic-type’ responses [4], in that they are seen in response to both human and non-human infants. Lorenz [1] proposed that infant features are ‘social releasers’, innate characteristics that lead to a care-giving response, usually from the parent. According to this view, human adults are hard-wired to respond to infant features in human infants. This is adaptive as it increases the chances of offspring survival. However, the same set of social releasers may exist in the young of other species and possession of these traits in animals can result in the animal receiving care-giving behaviour from humans [3,5]. In humans, ‘cute’ infants receive higher quality maternal care, stimulate greater feelings of sympathy and are perceived as having a more positive temperament than less visually appealing infants [6,7]. Similarly, animals possessing these infant features elicit care-giving responses and inhibit aggression [8]. In addition, Nittono et al. [9] found increased careful motor behaviour in a global–local letter task in human adults that had viewed images of cute animals. Increased careful behaviour around animals may increase the animal’s chances of survival. Infant features can also enhance companion animal survival via eliciting adoption. For example, dog breeds with infant features, such as toy breeds, are more likely to be adopted from rescue shelters [10]. In addition, Waller et al. [11] found that dogs who produced a high frequency of inner eyebrow raises, which increases the size of the eyes relative to their face, were adopted more quickly from shelters compared to other dogs. While same-species alloparenting occurs among primates, cross-species alloparenting is rare and is typically a result of human intervention. In addition, while cases of humans being adopted by animals are reported, evidence to support this is sparse [12]. This makes the human-companion animal relationship unique. 

Despite the beneficial effects of infant features in pets on human–animal interactions, companion animal cruelty is a common occurrence. For example, in 2018 alone, the Royal Society for the Prevention of Cruelty to Animals (RSPCA) investigated 130,700 animal cruelty complaints [13]. Varying levels of animal abuse have been reported globally. For example, Vaughn et al. [14] reported a prevalence of 1.8% in a nationally representative US sample. However, participants were simply asked if they had ever been cruel to an animal and the nature and frequency of these incidents is, therefore, unclear. In an Italian sample, Baldry [15] reported an animal abuse prevalence of 50.8% in children aged 9 to 17. In this study however, a distinction was made between different types of abuse from ‘bothering’ animals to ‘tormenting’ and ‘being cruel’. This suggests that low-level animal abuse may be relatively common. Indeed, Parfitt and Alleyne [16] found that of 150 English University students, over 60% endorsed animal abuse to some extent by reporting some level of positive feeling towards animal abuse scenarios. 

Animal welfare researchers are exploring potential correlates of animal abuse with the aim of identifying risk factors and prevention strategies for such behaviour [17,18]. One such correlate may be attitude towards animals. Attitudes towards animals are typically measured by assessing the acceptability of animal use to participants. Attitudes towards animals may depend on the species of animal and their similarity to humans [19]. In addition, the context of the animal use is important. For example, animal attitude questionnaires include questions on use of animals for food, medical research and for economic gain, in addition to causing intentional harm (e.g., [20,21,22]). Individuals can adopt a deontological animal-rights stance where animal use of any kind is viewed as morally wrong. Others may adopt a more centrist utilitarian stance where animal use is perceived as acceptable if the means justify the ends [23]. In contrast, from the contractarian perspective, animal suffering is not important in and of itself, but only if it has a negative effect on human wellbeing [24]. Attitude towards animals are associated with measures of human-directed empathy and attachment to pets [25,26,27]. Furthermore, associations have been identified between attitudes towards the treatment of animals and engagement in animal abuse [22]. Therefore, attitudes towards animals may be a useful proxy for a propensity to participate in acts of animal abuse.

There is also evidence to suggest that animal abuse is associated with the possession of psychopathic traits; Psychopathy is characterised by attributes such as a lack of empathy and remorse, deceitful and manipulative behaviour, superficial charm and an inflated sense of self [28]. Psychopathy can be broken down into two distinct constructs, Primary psychopathy and Secondary psychopathy [29]. Primary psychopathy is thought to have a significant genetic component and is associated with planned, deliberate action and a lack of emotional responses [30,31,32]. On the other hand, Secondary psychopathy is thought to be a consequence of environmental factors such as a history of maltreatment, and is characterised by deficits in impulse control [31,32,33]. Importantly, in contrast to Primary psychopathy, Secondary psychopaths are thought to have the ability to feel remorse and fearfulness [34]. Therefore, Primary psychopathic traits are argued to reflect ‘true’ psychopathy, while Secondary traits may be indicative of not only psychopathy, but also several other disorders such as ADHD and borderline personality disorder [34,35]. While psychopathy is thought to affect 1%–3% of the general population, psychopaths make up a significant proportion of prison inmates [36] and individuals with psychopathic traits may be more likely to engage in animal abuse than the general population. For example, Stupperich and Strack [37] found the psychopathic traits of lack of remorse, lack of empathy, superficiality and grandiosity to be characteristic of animal abusers in a German forensic patient sample. Indeed, a lack of empathy in particular has been suggested as a key psychological mechanism relating to the performance of animal abuse [17]. One limitation of past research is the tendency to focus on psychopathy in forensic samples [38]. Cases of animal abuse occur at a reasonably high rate in society in general [14,39]. Therefore, research with community samples can be beneficial for examining subclinical psychopathy and its correlates [31].

Two key theories of psychopathy have been put forward in recent years. The first is that psychopathy is associated with deficits in the ability to recognise facial cues, vocal cues, and body postures indicative of fear and sadness [40,41]. Similar to the process of emotion recognition, recognition of infant features in companion animals and human infants involves assessing facial cues, such as size of the eyes in relation to the head, and vocal cues, such as distress cries [3,42,43]. In keeping with the recognition deficit theory of psychopathy, it is possible that people with psychopathic traits may have deficits in their ability to recognize infant features, a key sign of vulnerability. This could go some way to explaining their increased propensity for violence against animals, particularly domesticated animals that retain infant features into adulthood [44]. However, there is evidence to suggest that individuals with psychopathic traits have an increased ability to recognise vulnerability [45]. For example, Wheeler, Book, and Costello [46] covertly recorded a number of female participants that later identified themselves as having been victimised or not victimised in the past. Male participants then rated the female participants’ gait for perceived vulnerability. In addition, male participants completed a self-report psychopathy test. Wheeler et al. [47] found that there was an association between the perceived vulnerability of a female participant and self-reported victimisation. In addition, there was an association between primary psychopathy score in male participants and victim identification accuracy. This finding has been replicated more recently (e.g., [47,48]) and is in apparent contrast to the principles underpinning Blair et al.’s [40] theory. An alternative theory of psychopathy put forward by Dadds et al. [49] states that psychopathy is brought about, not by a failure in recognising emotions, but by a failure in attending to the emotions of others. For example, eye contact with attachment figures is reduced in young children with high levels of callous–unemotional traits [50]. Based on the cumulative evidence, Viding and McCrory [51] conclude that individuals with psychopathic traits are able to understand the mental states of others. However, they are only likely to consider the perspective of others when it is self-benefiting and they tend to use this understanding for manipulation [51]. This makes sense from an evolutionary point of view; it has been theorised that personality traits that are viewed as ‘undesirable’ in society, may be adaptive in particular environments and a number of authors have suggested that psychopathy may be an adaptation rather than a pathological condition (e.g., [52,53]). For instance, in an environment composed largely of altruistic individuals, non-reciprocity and aggression could be effective tools for obtaining resources such as food or mates [54]. In such scenarios, a lack of empathy or remorse would make obtaining such resources easier [52]. Infant facial features can be thought of as an adaptation for survival in defenseless and naïve individuals [55]. An ability in non-reciprocators to recognise cuteness cues would have been beneficial for identifying susceptible targets for manipulation. This, coupled with reduced attentional priority to infant features, may have resulted in recognition, without the typically associated responses to such stimuli such as decreased aggression.

The aim of this research is to provide proof of concept in determining whether the ability to recognise and attend to infant features is linked to personality traits and attitudes towards animals in a community sample. Study 1 assesses associations between psychopathic traits and attitudes towards animals, and the ability to recognise cuteness cues via a two-alternative forced-choice task. Study 2 assesses associations between psychopathy and attitudes towards animals, and attention to ‘cute’ stimuli via a free-viewing eye-tracking task. If psychopathy is associated with deficits in the ability to recognise emotions [40], and this extends to recognition of infant features, we would expect individuals high in psychopathic traits to perform worse in the forced-choice task than those low in psychopathic traits. If psychopathy is brought about by a failure to attend to emotional cues [49], and this extends to attention to infant features, we would expect no difference in performance in the cuteness forced-choice task but decreased attention to cuteness cues in the free-viewing task by individuals with higher levels of psychopathy.

Hyp1: There will be no difference in forced-choice task scores between individuals with a High Primary Psychopathy score and Low Animal Attitude score (HP-LAA) and those with a Low Primary Psychopathy and High Animal Attitude score (LP-HAA).

Hyp2: HP-LAA individuals will give less attentional priority to ‘cute’ stimuli in a free-viewing task than LP-HAA individuals.

## 2. Study 1 Materials and Methods 

### 2.1. Ethical Statement

Ethical approval was obtained from the Queens University Belfast EPS Faculty Ethics Committee (EPS 19_120). All participants gave written informed consent. 

### 2.2. Demographic Information and Questionnaires 

Participants were recruited via posters/social media and via Prolific^®^ (Version: December 2019, Oxford, UK) an online participant recruitment platform for academics. Each participant recruited via Prolific^®^ was paid £1.25 to reward participation. Questionnaires completed in under 4 min were rejected. Demographic information was collected from each participant including age group, gender, occupation, pet-ownership status and parental-status. The 20-item Animal Attitudes Scale (AAS) [20] was used to assess participant attitudes towards animals. Each item is scored from 1 to 5, with a higher score being indicative of a pro-animal welfare attitude [20]. The AAS has a Cronbach’s alpha of between 0.85 to 0.95 [56]. The 26-item Levenson Self-Report Psychopathy Scale (SRPS) [57] was used to assess participants on Primary and Secondary psychopathic traits. Each item is scored from 1 to 5, with a higher score being more indicative of psychopathy. Mean scores for Primary and Secondary psychopathy were created by averaging responses from 16 and 10 of the questions respectively. The Levenson SRPS has a Cronbach’s alpha of 0.82 for primary psychopathy and 0.63 for secondary psychopathy [56]. A number of questions on both questionnaires were reverse scored.

### 2.3. Cuteness Forced-Choice Task

**Figure 1:**
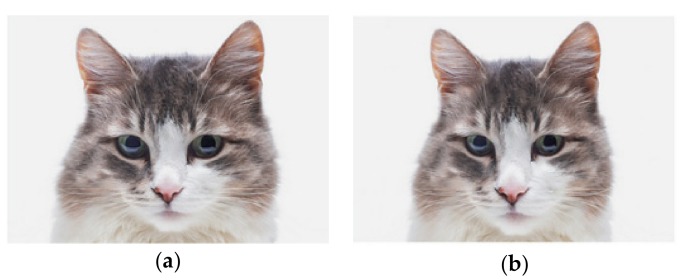
Examples of (**a**) high infant features (‘cute’); (**b**) low infant features (‘less cute’) version of the same animal photos: Thinkstock^®^/Getty Images^®^ (modified) [2], used in the cuteness forced-choice task.

Participants completed a cuteness forced-choice task online. Twelve image pairs of human infants (n = 4), cats (n = 4) and dogs (n = 4) were presented. Each pair consisted of two versions of the same animal or human, which varied in the extent to which they possessed infant features. Therefore, there was one ‘low infant features’ and one ‘high infant features’ version of each image. Pre-altered images were obtained with permission from Borgi et al. [2] (modified from Thinkstock/Getty Images, see Figure 1). Participants were presented with each image pair and required to choose which they deemed ‘cuter’. Participants were advised that they should make their choice as quickly as possible [58]. Image position (left or right) for each image pair was alternated between participants and image pair presentation sequence was randomised. The number of images correctly identified as being the ‘cuter’ image was calculated. Participants could score a minimum of 0 and a maximum of 12 points. Time to complete measures of ‘time to first click’, ‘time to last click’, ‘time to submit page’ and ‘average click count’ were recorded for each image pair per participant.

### 2.4. Statistical Analysis

Demographic information was analysed using SPSS version 24. A negative binomial regression was used to assess the ability of predictor variables: age group, gender, pet-ownership status, parental status, AAS and Primary and Secondary Levenson SRPS scores to predict the Dependent Variable: Cuteness forced-choice task score. A set of linear regression analyses were used to assess the ability of predictor variables: AAS and Primary and Secondary Levenson SRPS scores, to predict the Dependent Variables: ‘time to first click’, ‘time to last click’, ‘time to submit page’ and ‘average click count’. Pearson’s correlations were used to assess associations between AAS and Primary and Secondary Levenson SRPS scores.

## 3. Results

### 3.1. Demographic Information

Participant (n = 387) demographics can be seen in Table 1. There was a mean Primary psychopathy score of 2.05 (±0.44) and a mean Secondary psychopathy score of 2.36 (±0.47). Minimum and maximum scores for Primary and Secondary psychopathy were 1.0 and 3.3, and 1.0 and 4.0, respectively. There was a mean AAS score of 73.7 (±11.4). The minimum and maximum scores were 24 and 100, respectively.

### 3.2. Associations between Psychopathic Traits and Attitudes towards Animals

There was a moderate association between Primary and Secondary Levenson SRPS scores (r = 0.463, *p* < 0.001), a modest negative association between the AAS and Primary psychopathy score (r = −0.334, *p* < 0.001) and a weak negative association between AAS and Secondary Psychopathy score (r = −0.120, *p* = 0.020).

### 3.3. Predictors of Cuteness Forced-Choice Score

None of the variables predicted the forced-choice task score (*p* > 0.05). The mean identification accuracy was at 75%. Of all participants, 0.6% had a choice accuracy score of 25% or less.

### 3.4. Time to Complete

Primary and Secondary psychopathy score and AAS did not predict ‘time to first click’, ‘time to last click’, ‘time to submit page’ or ‘average click count’ (*p* > 0.05). Cuteness forced-choice task score was not associated with any time to complete measure (*p* > 0.05). Pet-ownership was a significant predictor of ‘time to first click’ (*p* = 0.005), ‘time to last click’ (*p* < 0.001) and ‘time to submit page’ (*p* < 0.001) but not ‘average click count’ (*p* > 0.05), with current pet-owners completing the task more quickly than those that have previously owned a pet or never owned a pet.

## 4. Study 2 Materials and Methods 

Study 2 assesses associations between psychopathy and attitudes towards animals, and attention to ‘cute’ stimuli via a free-viewing eye-tracking task.

### 4.1. Screening Process

The Animal Attitudes Scale [20] and Levenson Self-Report Psychopathy Scale (SRPS) [57] were completed online between 4 October and 18 November 2019. Participants (n = 142) were recruited via advertising the study on campus, and via social media and public notice boards. The same demographic information was collected for each participant as in study 1. In order to encourage participants with desirable characteristics to take part in study 2, a £20 reward for participation was offered. Due to financial constraints, a maximum of 50 participants were recruited from the original sample. This meant that AAS and SRPS scores could not be treated as continuous variables, and instead, a selection of participants with particularly high or low scores were selected for the eye-tracking study. A Psychopathy-Attitude Composite was created to identify suitable candidates for the free-viewing task by Tranforming the Levenson SRPS Primary Psychopathy scores and AAS score into z-scores. Primary psychopathy scores rather than Secondary psychopathy scores were selected as it has been argued that individuals with Secondary psychopathic traits are not necessarily psychopaths. Consequently, we focused on Primary psychopathy scores, which reflect a lack of empathy and the manipulation and exploitation of others [34,59]. In addition, the validity and reliability of results may be improved when considering Primary and Secondary psychopathic traits as distinct [30]. The AAS questionnaire was reverse scored. The z-scores for Levenson SRPS Primary Psychopathy and AAS were then added together and divided by two, giving both measures equal weighting. The Psychopathy-Attitude Composite score was then divided into quartiles. Participants from the upper and lower quartiles were invited to participate in the free-viewing task, creating two groups; High Primary Psychopathy–Low Animal Attitude Score (HP-LAA) and Low Primary Psychopathy–High Animal Attitude Score (LP-HAA). The final sample size for the Free-viewing task was n = 50 (HP-LAA, n = 21, LP-HAA, n = 29). See Table 2 for a summary of participant scores by group. The mean Primary psychopathy score was higher in HP-LAA and LP-HAA individuals than approximately 66% and 29% of the general population respectively. In addition, the Secondary psychopathy scores were higher in HP-LAA and LP-HAA individuals than 54% and 25% of the general population respectively [60]. Norms for the AAS were unavailable (Hal Herzog, personal communication). 

### 4.2. Design

This study employed a 2 (Psychopathy-Attitude Composite: High Primary Psychopathy–Low Animal Attitude (HP-LAA) vs. Low Primary Psychopathy –High Animal Attitude Score (LP-HAA)) × 4 (image category: Cute vs. Neutral vs. Monetary vs. Control) factorial design. Psychopathy-Attitude Composite was a between subjects factor and image category was a within subjects factor.

### 4.3. Free-Viewing Task: Image Characteristics

Four Image Categories were created using images from the International Affective Picture System (IAPS) database [61]; ‘Cute’ images of infant animals and babies of high valence (image No. 1410, 1440, 1441, 1460, 1463, 1710, 1722, 1750, 2045, 2050, 2070, 2071, 2075, 2080, 2151), ‘Neutral’ images of people and animals of neutral valence (image No. 1121, 1350, 1390, 1675, 1908, 1935, 2002, 2056, 2191, 2382, 2383, 2394, 2396, 2397, 2749), ‘Monetary’ images of money, gold, jewellery and sports cars of neutral to high valence (8500, 8501, 8502, 8503, 8510, 8531) and ‘Control’ images of inanimate objects such as a fire hydrant, tools and mop and bucket of neutral valence (image No. 7011, 7014, 7019, 7026, 7032, 7033, 7039, 7041, 7042, 7052, 7057, 7061, 7078, 7081, 7100). Arousal scores for images in each category were low to neutral. However, one-way analysis of variance (ANOVA) with least significant difference (LSD) post-hoc revealed that there was a significant difference in mean image arousal score between all image categories with Monetary being highest (5.24 ± 0.58), followed by Cute images (4.70 ± 0.49), Neutral Images (3.95 ± 0.63) and Control images (3.43 ± 0.48, *p* < 0.001). Supplementary ‘Monetary’ images were required and valence and arousal scores were needed for these images. In addition, while all ‘Cute’ images scored highly for valence within the database, they may not have necessarily been deemed ‘cute’ by the raters and may have been attractive for other reasons. Therefore, a panel of raters were needed to assess the perceived ‘cuteness’ of these images. To ensure that differences in image complexity did not affect attention, image complexity was assessed and balanced across groups.

#### 4.3.1. Subjective Rating of Image Characteristics

##### Image ‘Cuteness’

A panel of 37 undergraduate psychology students rated images online in exchange for course credits. Cuteness was assessed in images from the ‘Cute’ and ‘Neutral’ image categories as both contained images of humans and animals. For the cuteness rating task, raters were required to rate images from 1 (not at all cute) to 9 (very cute). Cuteness was defined as ‘being pleasant and attractive in a pretty or endearing way, especially of something or someone small or young’, adapted from Oxford and Cambridge dictionary definitions.

Participants were given the following instructions for rating the cuteness of each image; ‘At one extreme of the cute vs. not cute scale, you found the image attractive, pretty, endearing or pleasant. At the other end of the scale you found the image unattractive, unaesthetic, unappealing, repulsive’. A *t*-test revealed that ‘Cute’ images (7.88 ± 0.42) were rated higher for cuteness than ‘Neutral’ images (4.27 ± 0.84, *p* < 0.001). Twelve supplementary images were obtained from royalty-free image databanks to make up the ‘Monetary’ image category. Participants were asked to rate these images, along with 13 filler images, for valence and arousal as per the IAPS protocol [61] but adapted for completion online. The mean rating for ‘Monetary’ images was 5.01 (0.27). Therefore, they were rated as being neutrally valenced. ‘Monetary’ images from the IAPS database were rated within the database as high valence. Therefore, ‘Monetary’ images varied in rated valence. The potential implications of this are included in the discussion section of this paper.

##### Image Complexity

For the image complexity rating task, raters were required to rate images from 1 (not at all complex) to 9 (extremely complex). Raters were instructed to consider an image as being complex either because it had; (a). many simple objects that each had little detail, or (b). a few objects, with each being very detailed [62]. One-way ANOVA with LSD post-hoc revealed that there was a difference in image complexity between the groups. Specifically, images in the ‘Neutral’ treatment group were rated as being more complex than images from the other treatment groups (*p* = 0.001). Consequently, a number of images were replaced. A second rating panel composed of 14 psychology undergraduate students assessed the complexity of the new set of images. After the second rating session, a one-way ANOVA revealed that there was no difference in complexity rating for Cute (4.74 ± 0.95), Neutral (4.78 ± 0.98), Monetary (4.92 ± 0.68) or Control (4.62 ± 1.07) images (*p* > 0.05). Differences in image complexity between the four Image Categories were also assessed objectively by comparing the number of bytes in each image file after compression using a one-way ANOVA. Files of a larger byte size were interpreted as being more complex [62,63,64]. There was no significant difference in image complexity across the Cute (42.66 ± 16.57), Neutral (47.23 ± 11.57), Monetary (46.47 ± 19.29) and Control (48.41 ± 16.19) groups (*p* > 0.05).

### 4.4. Free-Viewing Task: Apparatus

Gaze data was collected using a Remote Eyetracking Device (RED) (SensoMotoric Instruments GmbH (SMI), Teltow, Germany). The iView X™ (Inition, London, UK) dark pupil tracking system with infrared illumination was used to track corneal reflexes. Experiment Center™ 3.0 (SensoMotoric Instruments GmbH (SMI), Teltow, Germany) was used to design the study and BeGaze™ (SMI, Teltow, Germany) software was used to produce and visualise the data from the study.

### 4.5. Free-Viewing Task: Data Reduction

BeGaze™ software recorded all monocular and binocular eye-tracking data for each participant so that it could be computed and analyzed. When analysing the eye-tracking data, an area of interest (AOI)-based approach was used. AOI’s define regions on the stimulus to quantify whether or how often each participant looks at that region [65]. Using the ‘AOI editor tool’, 60 AOIs (15 ‘Cute’, 15 ‘Neutral’, 15 ‘Monetary’ and 15 ‘Control’) were created, which fully covered each image. The spatial distribution of visual attention within each AOI was quantified using three dimensions of gaze behaviour; duration, frequency, and ordering. Duration was measured using the eye-tracking metrics dwell time (ms), average fixation duration (ms) and first fixation duration (ms). Frequency of gaze behaviour was measured using fixation count (ms) and ordering of gaze behaviour was measured using sequence (the order of gaze hits into the AOI based on entry time).

### 4.6. Free-Viewing Task: Procedure

Data collection took place between 13 November and 11 December 2019. Participants were escorted to a waiting area and issued with a participant information sheet. Participants read and initialled a consent form and were then escorted into an artificially lit test room comprised of a table, chair and the eye-tracking laptop computer. Participants were seated 60 to 80 cm from the eye-tracker and were encouraged to minimise head movements. Before each eye-tracking session, each participant completed a 9-point calibration procedure. The accuracy of the calibration was validated by visually comparing the participant gaze positions to the calibration targets, repeating the calibration procedure where necessary. Instructions were then presented on-screen. Participants were instructed to view 30 pairs of images as if they were watching television, and to focus on the fixation point between image presentations [66]. Fixation points were presented for 2000 ms and image pairs were presented for 3000 ms. Image position (left or right) for each image pair was alternated between participants and image pair presentation sequence was randomised. After the final image was presented, a text screen appeared thanking participants for their participation. Participants were then debriefed and paid £20 to reward participation.

### 4.7. Free-Viewing Task: Gaze Data

The following data were extracted for each image; dwell time (ms), first fixation duration (ms), fixation count, average fixation duration (ms) and sequence. Proportion of initial orientations towards images from each treatment were calculated from sequence data. If a participant only focused on one of the two presented AOIs, there would be no sequential output for that image pair. Data was excluded if more than 40% of the sequence data points per participant per image category were missing. Two different forms of attention were examined; First Fixation Duration and Initial Orientation measure involuntary attention, which is captured by one location or one aspect of the stimuli. Dwell time, average fixation duration and average fixation count capture those fixations that are actively and voluntarily exploring the stimuli, and their perceptual qualities, in much closer detail.

### 4.8. Statistical Analysis

Using a sample of 50 participants, there was 95% confidence, a standard error of 0.7 and confidence interval of 0.36 to 0.63. Mean scores were calculated from the 15 images per image category (Cute, Neutral, Monetary and Control) for each participant. A series of Pearson’s correlations were carried out to examine associations between raw AAS and SRPS scores and each individual eyetracking variable. A series of binary logistic regression analyses were then carried out to examine the contribution of predicator variables; proportion of initial orientations, mean dwell time, first fixation duration, average fixation duration and fixation count, in explaining Psychopathy-Attitude Composite classification (HP-LAA vs. LP-HAA). A series of mixed ANOVAs were used to examine interactions between Psychopathy-Attitude Composite classification (HP-LAA vs. LP-HAA) and image category (Cute vs. Neutral vs. Monetary vs. Control) on the proportion of initial orientations, mean dwell time, first fixation duration, average fixation duration and fixation count.

## 5. Results

See Table 3 for associations between raw AAS and SRPS scores and each individual eyetracking variable. See Table 4 for eye-tracking participant demographic information. 

### 5.1. Associations between Psychopathic Traits and Attitudes towards Animals before Participant Screening

There was a moderate association between Primary and Secondary psychopathy scores (r = 0.353, *p* < 0.001). There was a strong negative association between the Animal Attitudes Score and Primary psychopathy score (r = –0.534, *p* < 0.001) and a weak negative association between Animal Attitudes Score and Secondary Psychopathy score (r = −0.179, *p* = 0.020.)

### 5.2. Eye-Tracking Variables

#### 5.2.1. Proportion of Initial Orientations

**Figure 2:**
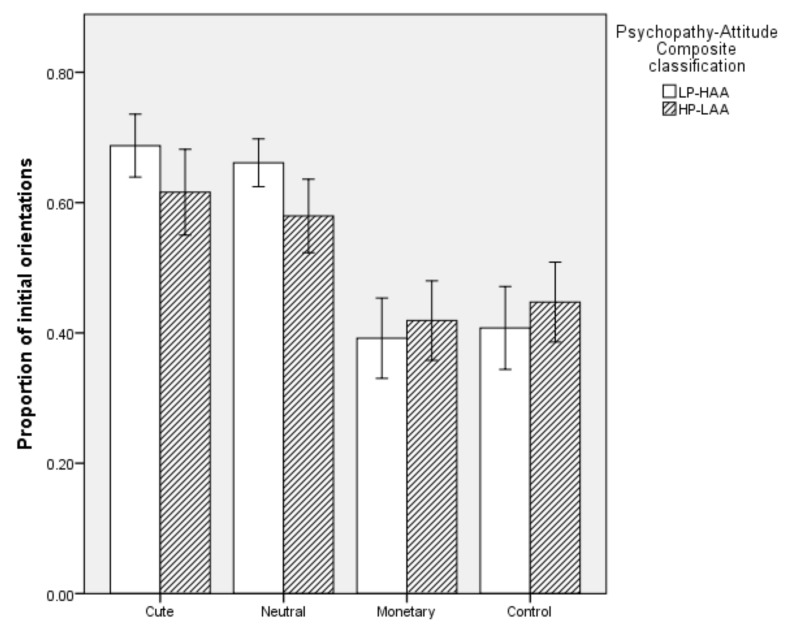
Proportion of initial orientations toward each image type in High Primary Psychopathy–Low Animal Attitude (HP-LAA) and Low Primary Psychopathy–High Animal Attitude (LP-HAA) participants.

Overall, the model was significant (*χ^2^* (2) = 9.873, *p* = 0.007). The model explained 28.9% of the variation in Psychopathy-Attitude Composite classification and correctly classifed 70.7% of cases. Specifically, HP-LAA participants had a lower proportion of initial oritentations towards Neutral images (*p* = 0.024) than LP-HAA participants. Proportion of initial orientations to Cute images did not predict Psychopathy-Attitude Composite classification. However, there was a trend towards a lower proportion of initial oritentations to Cute images in HP-LAA participants (*p* = 0.09). Proportion of initial orientations toward Monetary and Control images did not predict Psychopathy-Attitude Composite classification (*p* > 0.05, see Figure 2). 

#### 5.2.2. Dwell Time 

**Figure 3:**
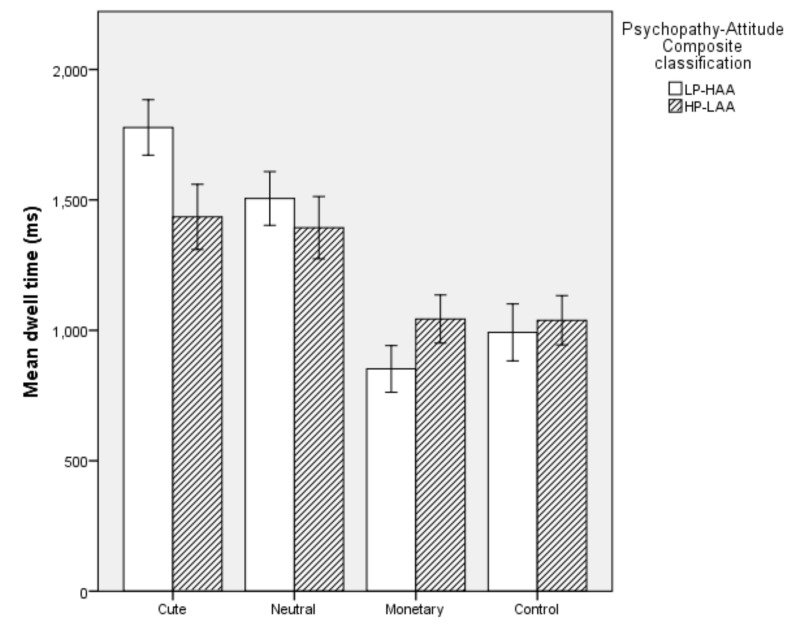
Mean dwell time on each image type in High Primary Psychopathy–Low Animal Attitude (HP-LAA) and Low Primary Psychopathy–High Animal Attitude (LP-HAA) participants.

Overall, the model was significant (*χ^2^* (2) = 20.563, *p* < 0.001). The model explained 45.4% of the variation in Psychopathy-Attitude Composite classification and correctly classifed 74% of cases. Specifically, HP-LAA participants had a decreased dwell time on Cute images (*p* = 0.003) and increased dwell time on Monetary images (*p* = 0.042) than LP-HAA participants. Dwell time on Neutral and Control images did not predict Psychopathy-Attitude Composite classification (*p* > 0.05, see Figure 3).

#### 5.2.3. First Fixation Duration

**Figure 4:**
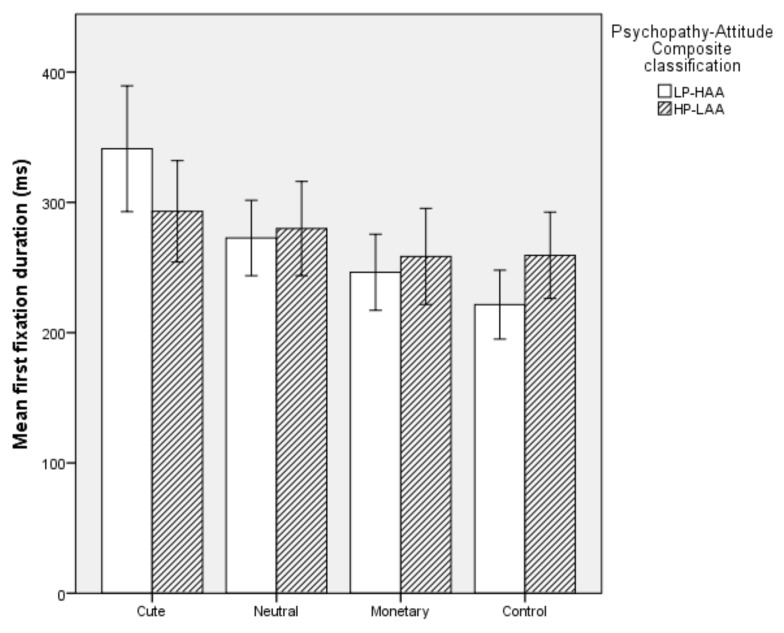
Mean first fixation duration on each image type in High Primary Psychopathy–Low Animal Attitude (HP-LAA) and Low Primary Psychopathy–High Animal Attitude (LP-HAA) participants.

Overall, the model was significant (*χ^2^* (2) = 7.321, *p* = 0.026). The model explained 18.3% of the variation in Psychopathy-Attitude Composite classification but did not correctly classify cases above chance level. Specifically, HP-LAA participants had an increased first fixation duration on Control images (*p* = 0.045). First fixation duration on Cute, Neutral and Monetary images did not predict Psychopathy-Attitude Composite classification (*p* > 0.05, see Figure 4).

#### 5.2.4. Mean Fixation Duration

**Figure 5:**
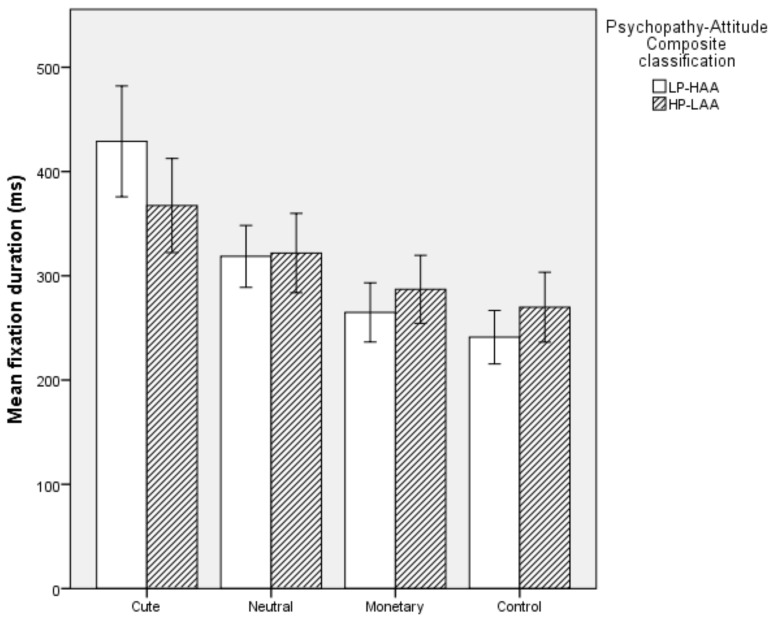
Mean fixation duration on each image type in High Primary Psychopathy–Low Animal Attitude (HP-LAA) and Low Primary Psychopathy–High Animal Attitude (LP-HAA) participants.

Overall, the model was significant (*χ^2^* (2) = 7.302, *p* = 0.026). The model explained 18.3% of the variation in Psychopathy-Attitude Composite classification and correctly classifed 68% of cases. Specifically, HP-LAA participants had a decreased mean fixation duration on Cute images (*p* = 0.036) and there was a trend towards increased mean fixation duration on Monetary images (*p* = 0.060). Mean fixation duration on Neutral and Control images did not predict Psychopathy-Attitude Composite classification (*p* > 0.05, see Figure 5).

#### 5.2.5. Mean Fixation Count

**Figure 6:**
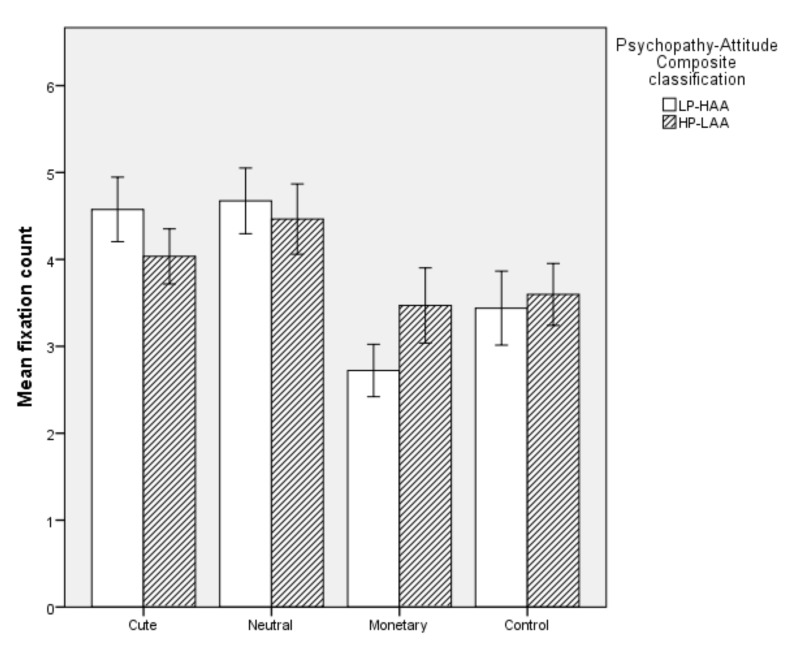
Mean fixation count on each image type in High Primary Psychopathy–Low Animal Attitude (HP-LAA) and Low Primary Psychopathy–High Animal Attitude (LP-HAA) participants.

Overall, the model was significant (*χ^2^* (2) = 20.625, *p* < 0.001). The model explained 45.5% of the variation in Psychopathy-Attitude Composite classification and correctly classifed 80% of cases. Specifically, HP-LAA participants had a decreased mean fixation count on Cute images (*p* = 0.005) and an increase in mean fixation count on Monetary images (*p* = 0.001). Mean fixation count on Neutral and Control images did not predict Psychopathy-Attitude Composite classification (*p* > 0.05, see Figure 6).

### 5.3. Interactions between Psychopathy-Attitude Composite Classification and Image Category

**Figure 7:**
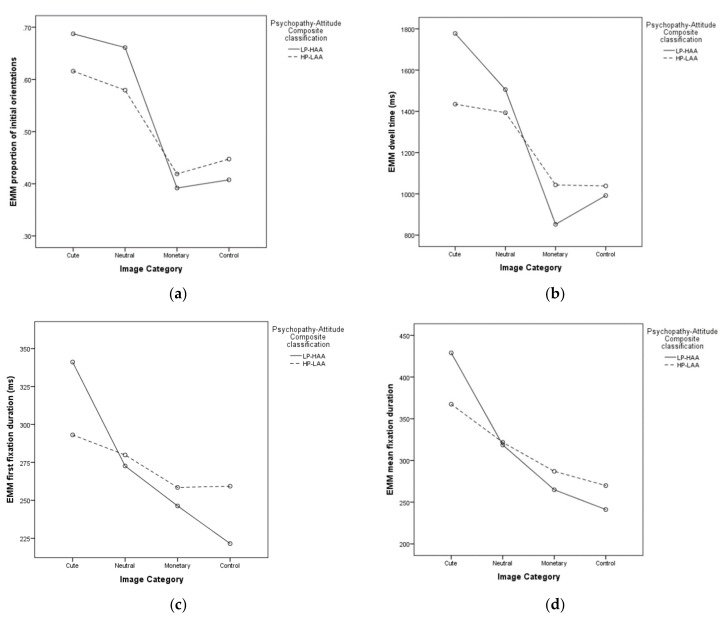

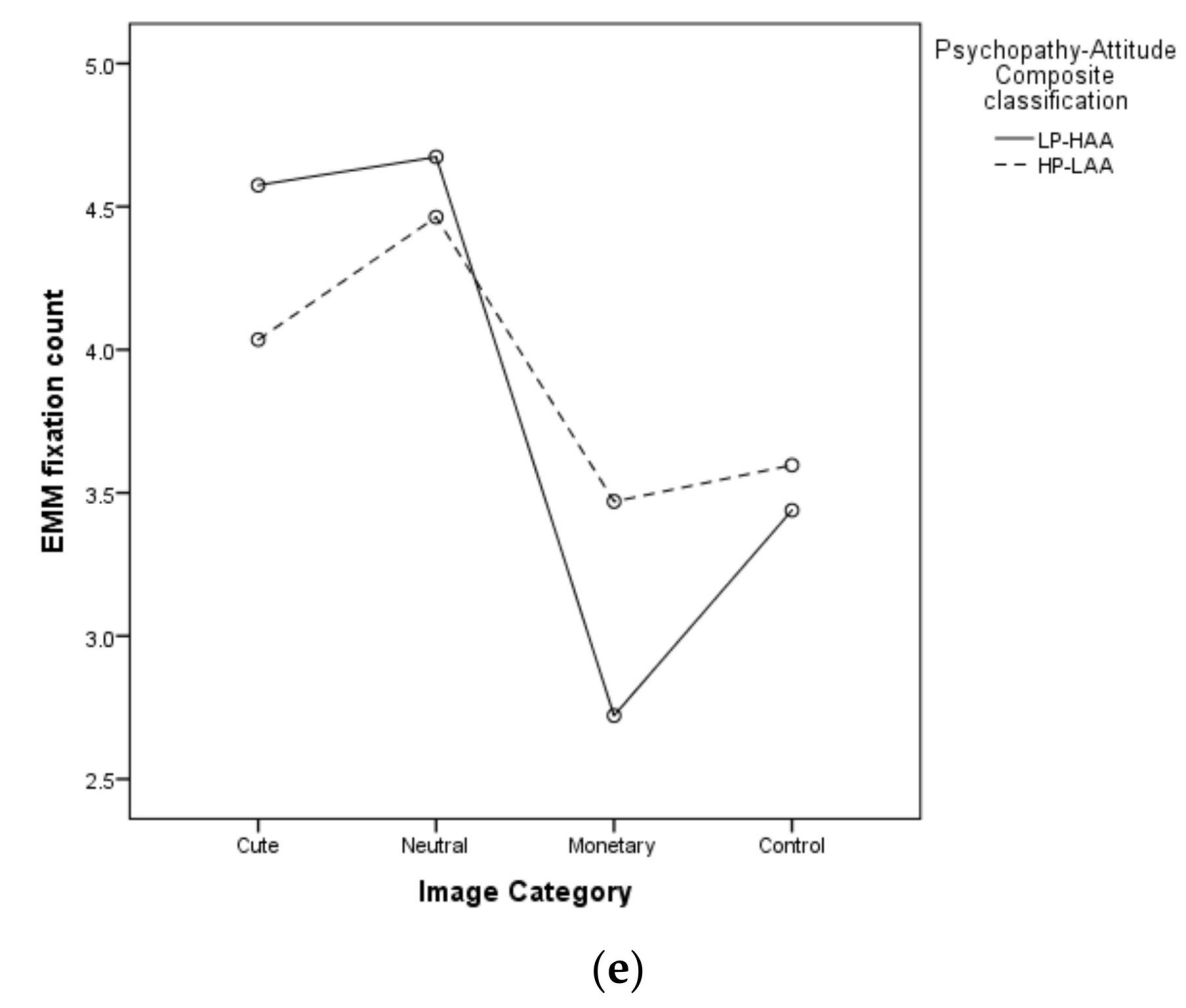
Estimated marginal means (EMM) for each eye-tracking variable (**a**–**e**) as a function of Psychopathy-Attitude Composite classification and image category.

There was a significant interaction between Psychopathy-Attitude Composite classification and image category in terms of dwell time (F (3) = 9.079, *p* < 0.001, Figure 7a), first fixation duration (F (3) = 3.667, *p* = 0.014, Figure 7b), mean fixation duration (F (3) = 4.103, *p* = 0.008, Figure 7c) and fixation count (F (3) = 7.964, *p* < 0.001, Figure 7d). There was no interaction effect between Psychopathy-Attitude Composite classification and image category in terms of proportion of initial orientations (*p* = 0.09, Figure 7e). However, numerically, the proportion of initial orientations followed a trend similar to that seen for the other eye-tracking variables.

## 6. Discussion

### 6.1. Relationships between Psychopathic Traits and Animal Attitudes

In both studies, primary and secondary psychopathy were moderately associated with each other. The strength of this association is analogous to the strength of association found by Levenson [57] when developing the scale for non-institutionalised populations. Animal attitude scores were moderately to strongly associated with Primary psychopathy and were weakly associated with Secondary psychopathy in both studies. Secondary psychopathic traits may be indicative of several disorders, other than psychopathy, that are not typically associated with aggression or callousness [34,67]. The moderate to strong association between primary psychopathy and animal attitudes may reflect the primary psychopathic trait of lack of empathy. For example, it has been argued that individuals with primary psychopathic traits lack the ability to empathize with others while those with secondary traits may perform antisocial behaviour but are more remorseful [34]. Several questions on the Animal Attitudes Scale may have tapped into the lack of empathy that is characteristic of primary psychopathy such as ‘I think people who object to raising animals for meat are too sentimental’, ‘I sometimes get upset when I see wild animals in cages at zoos’ and ‘The use of animals in rodeos and circuses is cruel’ [19]. In addition, individuals with primary and secondary psychopathic traits vary in the types of aggression shown, with primary psychopaths showing more premeditation in their aggressive acts and secondary psychopaths showing reactionary aggression [68]. Some of these differences could explain variation in attitudes towards animals in individuals high in primary versus secondary psychopathic traits. However, further research is needed to explore this further.

### 6.2. Cuteness Forced-Choice Task Scores and Attention to ‘Cute’ Stimuli

The ability to accurately select the cuter of each image pair was high across all participants and the results suggests that people with higher levels of psychopathic traits and poorer attitudes towards animals are just as able to recognise infant features as other individuals. Studies with a time limit on responses have identified deficits in emotional recognition in psychopaths while those without time limits tend to find no such deficits [46]. However, in the current study, while participants were instructed to choose an answer in the forced-choice task as quickly as possible, no time limit was imposed. ‘Time to respond’ data indicated that psychopathy score and animal attitudes scores were not associated with time taken to complete the forced choice task. Indeed, only 0.6% of participants had an accuracy of 25% of less in the forced choice task. This finding supports the theory that the ability to detect cuteness cues is widespread among human adults; The mean modern-day hunter–gatherer infant mortality rate is 26.8%, which suggests that within the Environment of Evolutionary Adaptedness (EEA), infant survival would likely have been at a similar level and, consequently, subject to strong selection pressures [55]. Infants that did not elicit adequate care would not survive to pass on their genes. In addition, infant features are thought to reflect infant quality in terms of genetic variation, immune response and longevity [69]. Consequently, adults that failed to direct care to ‘cuter’ offspring would have fewer surviving offspring than those that responded to infant features with increased caregiving behaviour. Within the EEA, it is likely that humans lived in small family groups [70]. Caregiving towards animals could then have arisen when the cost of failing to care for related young outweighed the costs of offering care to non-relatives [71]. This is evidenced in our nurturant behavioural responses to a key set of infant features that are characteristic not only of human infants, but of several species of animals [5,72]. The difference between non-psychopaths and those with psychopathic traits may therefore lie in the attention given to infant features; in the free viewing task, HP-LAA participants paid less attention to Cute stimuli in terms of dwell time, mean fixation duration and fixation count compared to LP-HAA participants. In addition, decreased attention to Neutral images and increased attention to Monetary and Control images were seen in HP-LAA participants compared to LP-HAA participants over a number of eye-tracking variables. Therefore, there was a reduction in attention to social features in general in HP-LAA participants. Similarly, Bedford et al. [73] found that lower preferential tracking of a face versus an inanimate object in 5-week-old infants was associated with the presence of callous-unemotional traits at 2.5 years of age.

Social orienting, i.e., responses to social cues such as faces [74], can be considered as a motivational behaviour [51]. Therefore, the findings of the current study could suggest that HP-LAA individuals had a reduced motivation to attend to social stimuli, including cuteness. Indeed, in the current study, the associations between Psychopathy-Attitude Composite classification and the assessed eye-tracking variables varied according to the type of attention that they reflected; the free viewing task can measure both voluntary and involuntary attention. Initial orientation and first fixation duration on an area of interest can be considered to be involuntary, while mean dwell time, mean fixation duration and mean fixation count reflect more voluntary attentional processes. Voluntary attention involves conscious direction of attention to an area containing information important to immediate goals. Considering this, voluntary attention is also known as goal-directed attention [75]. Involuntary attention, on the other hand, occurs when a stimulus captures attention even when it is unrelated to the current activity [75]. With regard to the Cute image category, all of the measures of voluntary attention were decreased in HP-LAA compared to LP-HAA participants while there was no association between Psychopathy-Attitude Composite classification and either of the involuntary attention measures. This is in line with the findings of study 1 that individuals with psychopathic traits are able to recognise infant features. It could be that involuntary attention to cute stimuli is hard-wired in humans to ensure the survival of one’s own offspring. However, individuals with psychopathic traits may then choose to give them less attention when there is no instrumental value in doing so. In general, participants tended to pay most attention to Cute and Neutral images and less attention to Monetary and Control images. However, the differences in attentional capture for the different Image Categories was much less pronounced in HP-LAA compared to LP-HAA participants. Indeed, a significant interaction effect was found between Psychopathy-Attitude Composite classification and image category for four of the five eye-tracking measures including first fixation duration, a measure of involuntary attention. In the cuteness forced-choice task, participants needed to attend to facial cues in order to perform well in the task. The ability to detect cuteness cues by HP-LAA participants in this case may reflect the instrumental use of social cues by psychopathic individuals to achieve an aim or goal [51]. In contrast, the free viewing task simply requires participants to view images as they wish, without the need to attend to anything in particular. In this scenario, there is no longer a need to focus on social cues such as cuteness. This could explain the voluntary attentional shift to Monetary and Control images. Indeed, HP-LAA individuals showed significantly more voluntary attention (dwell time, mean fixation duration and fixation count) to Monetary images compared to LP-HAA participants. It may be more beneficial for individuals with psychopathic traits to attend to stimuli associated with material resources when there is no benefit gained from attending to social cues.

### 6.3. Limitations and Future Directions

This proof of concept study was one of the first to apply theories of psychopathy to infant features in animals, and is an initial step in determining the future landscape for how individuals ‘at risk’ of committing animal abuse process information on infant features; a visual cue of vulnerability. However, there were a number of limitations. For example, a potential limitation of these studies is that the majority of participants scored relatively low on psychopathic traits and most had a positive attitude towards animals. This is to be expected as clinical psychopathy is thought to be present in only 1% to 3% of the general population [36]. However, the relatively large sample size in study 1 was sufficient for capturing a range of scores. Self-report measures allow researchers to screen a large pool of participants from community samples and individuals scoring highly on the trait of interest can then be recruited for further research [76]. Similar to Sethi et al. [33], a quartile split approach was used in the current study. While only 50 participants completed the eye-tracking task, the screening process allowed us to identify participants that varied considerably in their Levenson SRPS and AAS scores. A larger study with an increased sample size is now needed. This would allow psychopathy and animal attitude scores to be examined as separate dependent variables on a continuous scale. 

Another potential limitation of the study was the variation in image valance between image categories. The original intention was to have two high valence (Cute and Monetary) and two neutrally valenced (Neutral and Control) categories. However, most monetary images were rated as being neutrally valenced. Despite this, while being of neutral valence, Monetary images scored more highly on arousal compared to the other image categories. This could also have affected attention given to Monetary Images. According to the IAPS protocol, three pictures are shown to raters prior to rating to provide an example of the range of image types that will be rated. These often include a pleasant image, a neutral image, and a more distressing image such as a picture of a burn victim [61]. Therefore, it is likely that the valence scores gathered in the current study were lower for ‘Monetary’ images as the range from low to high valence was less extreme than that seen by raters of the IAPS images. In a meta-analysis of attention bias literature, Pool et al. [77] found that attention is biased toward positive or high-valence stimuli rather than neutral stimuli. In line with this, Cute images captured greater attention from both HP-LAA and LP-HAA participants compared to images from the other categories. Despite this however, an effect of Psychopathy-Attitude Composite classification was still seen. Future research could assess the effect of the possession of psychopathic traits on attention to Cute images compared to other high valence (e.g., erotic) or particularly low valence (e.g., mutilation or dead bodies) images [61]. 

Another potential limitation was that there were only four trials for each stimulus class (infants, cats and dogs) in study 1. This did not allow us to examine whether participant responses varied by species. A further study is needed that expands the range of images used. In addition, human infant and adult animal faces were used for study 1. Borgi and Cirulli [78] found that children show a preference for kittens and puppies over adult animals, prefer the faces of dogs over cats, and prefer puppies and kittens over human infants. Therefore, it would be of interest to include infant animals and human adult faces in future studies. It is also possible that this task was not complex enough to show any differences between participant groups. Future studies could use a signal detection paradigm where participants are shown a broader range of images, and individually indicate whether or not they are ‘cute’. 

In line with several previous studies, most participants were female [e.g., 29]. However, when participants were allocated to groups, it could be seen that there were 36.8% fewer male participants in the LP-HAA group compared to the HP-LAA group. This is in line with the finding that psychopathy and animal abuse are higher in males than females [79,80]. It remains uncertain as to whether any of the study participants have actually engaged in animal abuse. Further research should examine recognition of, and attention to infant features in individuals with a propensity for or history of animal abuse. In addition, in the current study, subclinical psychopathy was assessed via self-reporting. It is possible that the effects of the findings could be even more pronounced in clinically diagnosed psychopaths. Further research is needed to compare recognition of, and attention to infant features in community samples and individuals within forensic or clinical populations. Together, the findings from study 1 and study 2 suggest that individuals high in psychopathic traits and with poorer attitudes towards animals are able to recognise infant features but give them reduced attentional priority. This may have implications for animal welfare and warrants further investigation.

## Figures and Tables

**Table 1:** Study 1 participant demographic information.

Demographic Information	Percentage
**Recruitment route**	
Prolific^®^	85
Social media/posters	15
**County of residence**	
UK	78.8
USA	13.2
Other	8.0
**Gender**	
Female	70.3
Male	27.4
Other	1.6
Prefer not to say	0.3
**Age range**	
18–24	24.3
25–34	35.9
35–44	23.0
45–54	9.6
55–64	6.2
65–74	1.0
**Employment status**	
Employed	63.8
Unemployed	7.2
Student	18.6
Homemaker	8.3
Retired	2.1
Prefer not to say	1.8
**Pet ownership**	
Currently own a pet	61.2
Have never owner a pet	8.8
Previously owned a pet	30.0
**Parental status**	
Yes	33.6
No	65.6
Prefer not to say	0.8

**Table 2:** Study 2 participant scores by group.

Questionnaire	HP-LAA			LP-HAA		
Levenson SRPS	mean (±)	min	max	mean (±)	min	max
Primary Psychopathy	2.59 (0.65)	1.8	4.3	1.66 (0.37)	1.1	2.6
Secondary Psychopathy	2.61 (0.65)	1.8	3.9	2.07 (0.51)	1.1	3.4
Animal Attitude score	60.9 (13.10)	21	82	82.4 (8.39)	61	98

SRPS: self-report psychopathy scale.

**Table 3:** Associations between raw Levenson Self-Report Psychopathy Scale (SRPS) and Animal Attitude Scale scores, and each eye tracking variable.

Eyetracking Variable	Treatment	Levenson SRPS		Animal Attitude
		Primary	Secondary	
	Cute			
*p*^†^ of initial orientations		n.s.	n.s.	0.48 **
no. of fixations		−0.46 *	n.s.	n.s.
first fixation duration (ms)		n.s.	n.s.	n.s.
mean dwell time (ms)		−0.58 **	n.s.	n.s.
mean fixation duration (ms)		n.s.	n.s.	n.s.
	Neutral			
*p*^†^ of initial orientations		n.s.	0.46 *	0.34 *
no. of fixations		n.s.	n.s.	n.s.
first fixation duration (ms)		n.s.	n.s.	n.s.
mean dwell time (ms)		n.s.	n.s.	n.s.
mean fixation duration (ms)		n.s.	n.s.	n.s.
	Monetary			
*p*^†^ of initial orientations		n.s.	n.s.	n.s.
no. of fixations		n.s.	n.s.	n.s.
first fixation duration (ms)		n.s.	n.s.	−0.42 **
mean dwell time (ms)		n.s.	n.s.	−0.45 **
mean fixation duration (ms)		n.s.	n.s.	−0.33 *
	Control			
*p*^†^ of initial orientations		n.s.	n.s.	n.s.
no. of fixations		n.s.	n.s.	n.s.
first fixation duration (ms)		n.s.	n.s.	n.s.
mean dwell time (ms)		n.s.	n.s.	n.s.
mean fixation duration (ms)		n.s.	n.s.	n.s.

SRPS: self-report psychopathy scale. * *p* < 0.05. ** *p* < 0.01. ^†^ proportion.

**Table 4:** Study 2 participant demographic information by Psychopathy-Attitude Composite classification.

Demographic Information	HP-LAA	LP-HAA
**Gender**		
Female	61.9	72.4
Male	38.1	24.1
Other	0	3.4
**Age range**		
18–24	71.4	51.7
25–34	19.0	37.9
35–44	4.8	10.3
45–54	0	0
55–64	4.8	0
65–74	0	0
**Employment status**		
Employed	19	10.3
Unemployed	0	0
Student	81.0	89.7
Homemaker	0	0
Retired	0	0
**Pet ownership**		
Currently own a pet	28.6	65.5
Have never owned a pet	23.8	17.2
Previously owned a pet	47.6	17.2
**Parental status**		
Yes	4.8	10.3
No	95.2	89.7
Prefer not to say	0	0

HP-LAA: High Primary Psychopathy–Low Animal Attitude, LP-HAA: Low Primary Psychopathy–High Animal Attitude.

## References

[1] Lorenz K. (1943). Die angeborenen formen müglicher erfahrung (“The innate forms of potential experience”). Z. Tierpsychol..

[2] Borgi M., Cogliati-Dezza I., Brelsford V., Meints K., Cirulli F. (2014). Baby schema in human and animal faces induces cuteness perception and gaze allocation in children. Front. Psychol..

[3] Archer J. (1997). Why do people love their pets?. Evol. Hum. Behav..

[4] Prguda E., Neumann D.L. (2014). Inter-human and animal-directed empathy: A test for evolutionary biases in empathetic responding. Behav. Process..

[5] Archer J., Monton S. (2011). Preferences for Infant Facial Features in Pet Dogs and Cats. Ethology.

[6] Almanza-sepúlveda M.L., Dudin A., Wonch K.E., Steiner M., Feinberg D.R., Fleming A.S., Hall B. (2018). Infant Behavior and Development Exploring the morphological and emotional correlates of infant cuteness. Infant Behav. Dev..

[7] Parsons C.E., Young K.S., Bhandari R., Marinus H., Ijzendoorn V., Bakermanskranenburg M.J., Kringelbach M.L. (2014). The bonnie baby: Experimentally manipulated temperament affects perceived cuteness and motivation to view infant faces. Dev. Sci..

[8] Lehmann V., Huis in’t Veld E.M.J., Vingerhoets A.J.J.M. (2013). The human and animal baby schema effect: Correlates of individual differences. Behav. Process..

[9] Nittono H., Fukushima M., Yano A., Moriya H. (2012). The Power of Kawaii: Viewing Cute Images Promotes a Careful Behavior and Narrows Attentional Focus. PLoS ONE.

[10] DeLeeuw J.L. (2010). Animal Shelter Dogs: Factors Predicting Adoption Versus Euthanasia. Ph.D. Thesis.

[11] Waller B.M., Peirce K., Caeiro C.C., Scheider L., Burrows A.M., McCune S., Kaminski J. (2013). Paedomorphic facial expressions give dogs a selective advantage. PLoS ONE.

[12] Shipman P. (2010). The animal connection and human evolution. Curr. Anthropol..

[13] Royal Society for the Prevention of Cruelty to Animals (RSPCA). https://www.rspca.org.uk/whatwedo/latest/facts.

[14] Vaughn M.G., Fu Q., DeLisi M., Beaver K.M., Perron B.E., Terrell K., Howard M.O. (2009). Correlates of cruelty to animals in the United States: Results from the National Epidemiologic Survey on Alcohol and Related Conditions. J. Psychiatr. Res..

[15] Baldry A.C. (2003). Animal abuse and exposure to interparental violence in Italian youth. J. Interpers. Violence.

[16] Parfitt C.H., Alleyne E. (2018). Animal abuse proclivity: Behavioral, personality and regulatory factors associated with varying levels of severity. Psychol. Crime Law.

[17] Henry B.C. (2006). Empathy, home environment, and attitudes toward animals in relation to animal abuse. Anthrozoös.

[18] Oleson J.C., Henry B.C. (2009). Relations among need for power, affect and attitudes toward animal Cruelty. Anthrozoös.

[19] Batt S. (2009). Human attitudes towards animals in relation to species similarity to humans: A multivariate approach. Biosci. Horiz..

[20] Herzog H.A., Betchart N.S., Pittman R.B. (1991). Gender, sex role orientation, and attitudes toward animals. Anthrozoös.

[21] Henry B. (2004). The relation between animal cruelty, delinquency, and attitudes toward the treatment of animals. Soc. Anim..

[22] Henry B. (2004). Exposure to animal abuse and group context: Two factors affecting participation in animal abuse. Anthrozoös.

[23] Corrigan R.H., Farrell M.E. (2010). Ethics: A University Guide.

[24] Johansson-Stenman O. (2018). Animal Welfare and Social Decisions: Is It Time to Take Bentham Seriously?. Ecol. Econ..

[25] Hawkins R.D., Hawkins E.L., Williams J.M. (2017). Psychological Risk Factors for Childhood Nonhuman Animal Cruelty: A Systematic Review. Soc. Anim..

[26] Morton L., Daly B. (2017). Empathic Correlates of Witnessing the Inhumane Killing of an Animal: An Investigation of Single and Multiple Exposures. Soc. Anim..

[27] Signal T.D., Taylor N. (2007). Attitude to animals and empathy: Comparing animal protection and general community samples. Anthrozoös.

[28] Hare R.D., Hart S.D., Harpur T.J. (1991). Psychopathy and the DSM-IV Criteria for Antisocial Personality Disorder. J. Abnorm. Psychol..

[29] Lee Z., Salekin R.T. (2010). Psychopathy in a noninstitutional sample: Differences in primary and secondary subtypes. Personal. Disord..

[30] Del Gaizo A.L., Falkenbach D.M. (2008). Primary and secondary psychopathic-traits and their relationship to perception and experience of emotion. Personal. Individ. Differ..

[31] Falkenbach D., Poythress N., Creevy C. (2008). The exploration of subclinical psychopathic subtypes and the relationship with types of aggression. Personal. Individ. Differ..

[32] Wilkowski B.M., Robinson M.D. (2008). Putting the brakes on antisocial behavior: Secondary psychopathy and post-error adjustments in reaction time. Personal. Individ. Differ..

[33] Sethi A., McCrory E., Puetz V., Hoffmann F., Knodt A.R., Radtke S.R., Brigidi B.D., Hariri A.R., Viding E. (2018). Primary and Secondary Variants of Psychopathy in a Volunteer Sample Are Associated With Different Neurocognitive Mechanisms. Biol. Psychiatry Cogn. Neurosci..

[34] Dean A.C., Altstein L.L., Berman M.E., Constans J.I., Sugar C.A., McCloskey M.S. (2013). Secondary psychopathy, but not primary psychopathy, is associated with risky decision-making in noninstitutionalized young adults. Personal. Individ. Differ..

[35] Karpman B. (1941). On the need for separating psychopathy into two distinct clinical types: Symptomatic and idiopathic. J. Crim. Psychopathol..

[36] Johnson S.A. (2019). Understanding the violent personality: Antisocial personality disorder, psychopathy, & sociopathy explored. Forensic Res. Criminol. Int. J..

[37] Stupperich A., Strack M. (2016). Among a German Sample of Forensic Patients, Previous Animal Abuse Mediates Between Psychopathy and Sadistic Actions. J. Forensic Sci..

[38] Gao Y., Raine A. (2010). Successful and unsuccessful psychopaths: A neurobiological model. Behav. Sci. Law.

[39] (2020). Humane Society. https://www.humanesociety.org/resources/animal-cruelty-facts-and-stats.

[40] Blair R.J.R., Peschardt K.S., Budhani S., Mitchell D.G.V., Pine D.S. (2006). The development of psychopathy. J. Child Psychol. Psychiatry.

[41] Blair R.J.R. (2019). Dysfunctional neurocognition in individuals with clinically significant psychopathic traits. Dialogues Clin. Neurosci..

[42] McComb K., Taylor A.M., Wilson C., Charlton B.D. (2019). The cry embedded within the purr. Curr. Biol..

[43] Parsons C.E., Young K.S., Craske M.G., Stein A.L., Kringelbach M.L. (2014). Introducing the Oxford Vocal (OxVoc) Sounds database: A validated set of non-acted affective sounds from human infants, adults, and domestic animals. Front. Psychol..

[44] Trut L., Oskina I., Kharlamova A. (2009). Animal evolution during domestication: The domesticated fox as a model. Bioessays.

[45] Krupp D.B., Sewall L.A., Lalumière M.L., Sheriff C., Harris G.T. (2012). Nepotistic patterns of violent psychopathy: Evidence for adaptation?. Front. Psychol..

[46] Wheeler S., Book A., Costello K. (2009). Psychopathic traits and perceptions of victim vulnerability. Crim. Justice Behav..

[47] Book A., Costello K., Camilleri J.A. (2013). Psychopathy and Victim Selection: The Use of Gait as a Cue to Vulnerability. J. Interpers. Violence.

[48] Ritchie M.B., Blais J., Forth A.E., Book A.S. (2018). Identifying vulnerability to violence: The role of psychopathy and gender. J. Crim. Psychol..

[49] Dadds M.R., El Masry Y., Wimalaweera S., Guastella A.J. (2008). Reduced eye gaze explains “fear blindness” in childhood psychopathic traits. J. Am. Acad. Child Psychiatry.

[50] Dadds M.R., Allen J.L., Oliver B.R., Faulkner N., Legge K., Moul C., Woolgar M., Scott S. (2012). Love, eye contact and the developmental origins of empathy v. psychopathy. Br. J. Psychiatry.

[51] Viding E., McCrory E. (2019). Towards understanding atypical social affiliation in psychopathy. Lancet Psychiatry.

[52] Glenn A.L., Kurzban R., Raine A. (2011). Evolutionary theory and psychopathy. Aggress. Violent Behav..

[53] Jonason P.K., Webster G.D., Schmitt D.P., Li N.P., Crysel L. (2012). The antihero in popular culture: Life history theory and the dark triad personality traits. Rev. Gen. Psychol..

[54] Lalumière M.L., Harris G.T., Rice M.E. (2001). Psychopathy and developmental instability. Evol. Hum. Behav..

[55] Volk A.A., Atkinson J.A. (2013). Infant and child death in the human environment of evolutionary adaptation. Evol. Hum. Behav..

[56] Herzog H., Grayson S., Mccord D. (2015). Brief Measures of the Animal Attitude Scale. Anthrozoös.

[57] Levenson M.R., Kiehl K.A., Fitzpatrick C.M. (1995). Assessing psychopathic attributes in a noninstitutionalized population. J. Pers. Soc. Psychol..

[58] Lobmaier J.S., Sprengelmeyer R., Wiffen B., Perrett D.I. (2010). Female and male responses to cuteness, age and emotion in infant faces. Evol. Hum. Behav..

[59] Ferrigan M.M., Valentiner D.P., Berman M.E. (2000). Psychopathy dimensions and awareness of negative and positive consequences of aggressive behavior in a nonforensic sample. Personal. Individ. Differ..

[60] (2020). Open Psychometrics. Levenson Self-Report Psychopathy Scale. https://openpsychometrics.org/tests/LSRP.php.

[61] Lang P.J., Bradley M.M., Cuthbert B.N. (2005). International Affective Picture System (IAPS): Affective Ratings of Pictures and Instruction Manual.

[62] Calvo M.G., Lang P.J. (2004). Gaze patterns when looking at emotional pictures: Attention and affect. Motiv. Emot..

[63] Buodo G., Sarlo M., Palomba D. (2002). Attentional resources measured by reaction times highlight differences within pleasant and unpleasant, high arousing stimuli. Motiv. Emot..

[64] Yu H., Winkler S. Image complexity and spatial information. Presented at the 5th International Workshop on Quality of Multimedia Experience.

[65] Holmqvist K., Nyström M., Andersson R., Dewhurst R., Jarodzka H., Van de Weijer J. (2011). Eye Tracking: A Comprehensive Guide to Methods and Measures.

[66] Giel K.E., Friederich H.C., Teufel M., Hautzinger M., Enck P., Zipfel S. (2011). Attentional processing of food pictures in individuals with anorexia nervosa—An eye tracking study. Biol. Psychiatry.

[67] American Psychiatric Association (2013). Diagnostic and Statistical Manual of Mental Disorders (DSM).

[68] Anderson N.E., Kiehl K.A. (2014). Psychopathy: Developmental perspectives and their implications for treatment. Restor. Neurol. Neurosci..

[69] Franklin P., Volk A.A., Wong I. (2018). Are newborns’ faces less appealing?. Evol. Hum. Behav..

[70] Lewis D.M.G., Conroy-beam D., Asao K., Buss D.M. (2017). Evolutionary Psychology: A How-To Guide. Am. Psychol..

[71] Serpell J.A., Paul E.S., Salmon C., Shackelford T.K. (2012). Pets in the Family: An Evolutionary Perspective. The Oxford Handbook of Evolutionary Family Psychology.

[72] Golle J., Lisibach S., Mast F.W., Lobmaier J.S. (2013). Sweet Puppies and Cute Babies: Perceptual Adaptation to Babyfacedness Transfers across Species. PLoS ONE.

[73] Bedford R., Pickles A., Sharp H., Wright N., Hill J. (2015). Reduced Face Preference in Infancy: A Developmental Precursor to Callous-Unemotional Traits?. Biol. Psychiatry.

[74] Ramot M., Walsh C., Reimann G.E., Martin A. (2020). Distinct neural mechanisms of social orienting and mentalizing revealed by independent measures of neural and eye movement typicality. Commun. Biol..

[75] Prinzmetal W., Zvinyatskovskiy A., Gutierrez P., Dilem L. (2009). Voluntary and involuntary attention have different consequences: The effect of perceptual difficulty. J. Exp. Psychol..

[76] Hall J.R., Benning S.D., Patrick C.J. (2006). The “successful” psychopath. Handbook of Psychopathy.

[77] Pool E., Brosch T., Delplanque S., Sander D. (2016). Attentional Bias for Positive Emotional Stimuli: A Meta-Analytic Investigation. Psychol. Bull..

[78] Borgi M., Cirulli F. (2013). Children’s preferences for infantile features in dogs and cats. Hum. Anim. Interact. Bull..

[79] Alleyne E., Tilston L., Parfitt C., Butcher R. (2015). Adult-perpetrated animal abuse: Development of a proclivity scale. Psychol. Crime Law.

[80] Lobbestael J., van Teffelen M., Baumeister R.F. (2019). Psychopathy subfactors distinctively predispose to dispositional and state-level of sadistic pleasure. J. Behav. Ther. Exp. Psychiatry.

